# *N*-[4-Acetyl-5-(2-methylprop-1-enyl)-5-(2-*p*-tolyl­prop­yl)-4,5-dihydro-1,3,4-thia­diazol-2-yl]acetamide

**DOI:** 10.1107/S1600536809017127

**Published:** 2009-05-14

**Authors:** Noureddine Mazoir, Lahcen El Ammari, Nouzha Bouhmaida, Slimane Dahaoui, Ahmed Benharref, Moha Berraho

**Affiliations:** a Laboratoire de Chimie Biomoléculaires, Substances Naturelles et Réactivité, Faculté des Sciences Semlalia, BP 2390, Bd My Abdellah, 40000 Marrakech, Morocco; b Laboratoire de Chimie du Solide Appliquée, Faculté des Sciences, Avenue Ibn Battouta, BP 1014, Rabat, Morocco; c Laboratoire des Sciences des Matériaux, Département de Physique, Faculté des Sciences Semlalia, BP 2390, Bd My Abdellah, 40000 Marrakech, Morocco; d CRM2 (UMR UHP - CNRS 7036), Faculté des Sciences et Techniques, BP 70239, Bd des Aiguillettes, 54506 Vandoeuvre-lès-Nancy CEDEX, France

## Abstract

The title heterocyclic compound, C_20_H_27_N_3_O_2_S, was synthesized from 2-(4-methyl­cyclo­hex-3-en­yl)-6-methyl­hepta-2,5-dien-4-one, which was isolated from the essential oil *Cedrus atlantica*. The thia­diazole ring is essentially planar [maximum deviation 0.006 (2) Å] and it forms a dihedral angle of 18.08 (9)° with the benzene ring. The dihedral angle between the thia­diazole ring and the acetamide plane is 7.62 (10)°. In the crystal, mol­ecules are linked into chains running along the *c* axis by inter­molecular N—H⋯O hydrogen bonds.

## Related literature

For the biological activity of 1,3,4-thia­diazole derivatives, see: Demirbas *et al.* (2005 ▶); Holla *et al.* (2002 ▶); Kritsanida *et al.* (2002 ▶); Nizamuddin *et al.* (1999 ▶); Sun *et al.* (1999 ▶); Udupi *et al.* (2000 ▶). For the synthesis, see: Beatriz *et al.* (2002 ▶); Sakthivel *et al.* (2008 ▶). For related structures, see: Loughzail *et al.* (2009 ▶); Tebaa *et al.* (2009 ▶).
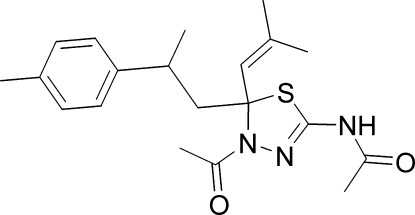


## Experimental

### 

#### Crystal data

C_20_H_27_N_3_O_2_S
*M* *_r_* = 373.51
Monoclinic, 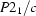

*a* = 10.855 (2) Å
*b* = 14.193 (2) Å
*c* = 12.854 (4) Å
β = 90.955 (11)°
*V* = 1980.1 (8) Å^3^
*Z* = 4
Mo *K*α radiation
μ = 0.18 mm^−1^
*T* = 100 K
0.28 × 0.17 × 0.12 mm

#### Data collection

Bruker X8 APEX CCD area-detector diffractometer
Absorption correction: none
7884 measured reflections
4030 independent reflections
3365 reflections with *I* > 2σ(*I*)
*R*_int_ = 0.032

#### Refinement

*R*[*F* ^2^ > 2σ(*F* ^2^)] = 0.044
*wR*(*F* ^2^) = 0.094
*S* = 1.10
4030 reflections
249 parameters
H atoms treated by a mixture of independent and constrained refinement
Δρ_max_ = 0.24 e Å^−3^
Δρ_min_ = −0.20 e Å^−3^

### 

Data collection: *APEX2* (Bruker, 2005 ▶); cell refinement: *SAINT-Plus* (Bruker, 2005 ▶); data reduction: *SAINT-Plus*; program(s) used to solve structure: *SHELXS97* (Sheldrick, 2008 ▶); program(s) used to refine structure: *SHELXS97* (Sheldrick, 2008 ▶); molecular graphics: *ORTEP-3 for Windows* (Farrugia, 1997 ▶) and *PLATON* (Spek, 2009 ▶); software used to prepare material for publication: *WinGX* (Farrugia, 1999 ▶).

## Supplementary Material

Crystal structure: contains datablocks I, global. DOI: 10.1107/S1600536809017127/ci2797sup1.cif

Structure factors: contains datablocks I. DOI: 10.1107/S1600536809017127/ci2797Isup2.hkl

Additional supplementary materials:  crystallographic information; 3D view; checkCIF report

## Figures and Tables

**Table 1:** Hydrogen-bond geometry (Å, °)

*D*—H⋯*A*	*D*—H	H⋯*A*	*D*⋯*A*	*D*—H⋯*A*
N1—H2⋯O2^i^	0.87 (2)	1.96 (2)	2.811 (2)	167 (2)

Symmetry code: (i) 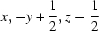.

**Fig. 1.**
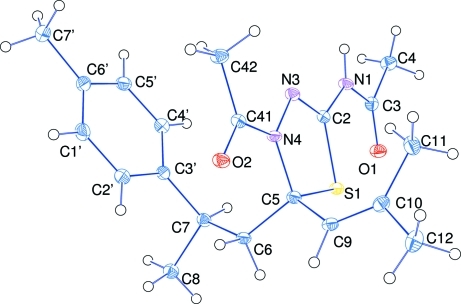
Molecular structure of the title compound, with the atom-labelling scheme. Displacement ellipsoids are drawn at the 30% probability level. H atoms are represented as small spheres of arbitrary radii.

**Fig. 2.**
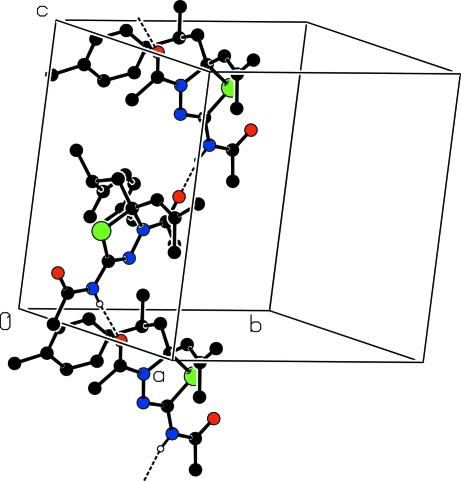
Partial packing view showing the N—H···O hydrogen bonds (dashed lines) and the formation of a chain along the c axis. H atoms not involved in hydrogen bonding have been omitted for clarity.

## supplementary crystallographic information

### Comment

1,3,4-Thiadiazole derivatives possess antimicrobial (Demirbas *et al.*, 2005) and antiviral (Kritsanida *et al.*, 2002) activities. They are also known for their broad-spectrum of biological activities such as antibacterial (Sun *et al.*, 1999), anti-inflammatory (Udupi *et al.*, 2000) and herbicidal (Nizamuddin *et al.*, 1999). In addition, [1,3,4]thiadiazoles exhibit various biological activities possibly due to the presence of the ═ N—C—S moiety (Holla *et al.*, 2002). In view of these findings and in continuation to our previous work on the synthesis of [1,3,4]thiadiazoles, we report herein the hemisynthesis of *N*-[4-acetyl-5-isobutenyl-5-(2-*p*-tolylpropyl)-4,5-dihydro-1,3,4-thiadiazol-2-yl]acetamide, (I), through chemical modification of 2-(4-methylcyclohex-3-enyl)-6-methylhepta-2,5-dien-4-one, which is isolated from Cedrus Atlantica essential oil. Thus, aromatization of this later, followed by condensation with thiosemicarbazide (Beatriz *et al.*, 2002; Sakthivel *et al.*, 2008) ending with treatment of acetic anhydride in the presence of pyridine yielded the diasterioisomers in high stereoselectivity.

The molecular structure of (I) is shown in Fig. 1. The geometric parameters (bond lengths and angles) are very similar to those observed in previously reported structures (Loughzail *et al.*,2009; Tebaa *et al.*,2009). The thiadiazole ring system is essentially planar and it forms a dihedral angle of 18.08 (9)° with the benzene ring.

In the crystal structure, molecules are linked into chains (Fig. 2) running along the *c* axis by intermolecular N—H···O hydrogen bonds (Table 1) involving the carbonyl and the acetamide groups.

### Experimental

A mixture of 2-(4-methylcyclohex-3-enyl)-6-methylhepta-2,5-dien-4-one (0.5 g, 2.3 mmol) and Pd/C (10%) was heated at 423 K for 12 h. The product obtained was treated with equimolecular quantity of thiosemicarbazide and several drops of HCl were added. The reactional mixture was heated at reflux in ethanol for 5 h and then evaporated under reduced pressure and the residue obtained was purified on silica gel column using hexane–ethyl acetate (96:4) as an eluent. 0.25 mmol of the thiosemicarbazone obtained was dissolved in 2.5 ml of pyridine and 2.5 ml of acetic anhydride. The mixture was heated on a water bath for 1.5 h. The resulting residue was concentrated *in vacuo* and chromatographied on silica gel column with hexane–ethyl acetate (92:8) as an eluent. Suitable crystals were obtained by evaporation of an ethyl acetate solution at 277 K.

### Refinement

Atoms H2 and H9 were located in a difference map and refined freely. The remaining H atoms were positioned geometrically and refined as riding, with C—H = 0.93 Å (aromatic), 0.96 Å (methyl), 0.97 Å (methylene), 0.98 Å (methine), and with *U*_iso_(H) = 1.2U_eq_(C) or 1.5U_eq_(C_methyl_).

### Crystal data

**Table:**

C_20_H_27_N_3_O_2_S	*F*(000) = 800
*M**_r_* = 373.51	*D*_x_ = 1.253 Mg m^−^^3^
Monoclinic, *P*2_1_/*c*	Mo *K*α radiation, λ = 0.71073 Å
Hall symbol: -P 2ybc	Cell parameters from 8068 reflections
*a* = 10.855 (2) Å	θ = 2.8–26.4°
*b* = 14.193 (2) Å	µ = 0.18 mm^−^^1^
*c* = 12.854 (4) Å	*T* = 100 K
β = 90.955 (11)°	Prism, colourless
*V* = 1980.1 (8) Å^3^	0.28 × 0.17 × 0.12 mm
*Z* = 4	

### Data collection

**Table:**

Bruker X8 APEX CCD area-detector diffractometer	3365 reflections with *I* > 2σ(*I*)
Radiation source: fine-focus sealed tube	*R*_int_ = 0.032
graphite	θ_max_ = 26.4°, θ_min_ = 2.8°
φ and ω scans	*h* = 0→13
7884 measured reflections	*k* = −17→17
4030 independent reflections	*l* = −16→16

### Refinement

**Table:**

Refinement on *F*^2^	Primary atom site location: structure-invariant direct methods
Least-squares matrix: full	Secondary atom site location: difference Fourier map
*R*[*F*^2^ > 2σ(*F*^2^)] = 0.044	Hydrogen site location: inferred from neighbouring sites
*wR*(*F*^2^) = 0.094	H atoms treated by a mixture of independent and constrained refinement
*S* = 1.10	*w* = 1/[σ^2^(*F*_o_^2^) + (0.0257*P*)^2^ + 1.5568*P*] where *P* = (*F*_o_^2^ + 2*F*_c_^2^)/3
4030 reflections	(Δ/σ)_max_ = 0.001
249 parameters	Δρ_max_ = 0.24 e Å^−^^3^
0 restraints	Δρ_min_ = −0.20 e Å^−^^3^

### Special details

**Table:**

Geometry. All esds (except the esd in the dihedral angle between two l.s. planes) are estimated using the full covariance matrix. The cell esds are taken into account individually in the estimation of esds in distances, angles and torsion angles; correlations between esds in cell parameters are only used when they are defined by crystal symmetry. An approximate (isotropic) treatment of cell esds is used for estimating esds involving l.s. planes.

### Fractional atomic coordinates and isotropic or equivalent isotropic displacement parameters (Å^2^)

**Table:**

	*x*	*y*	*z*	*U*_iso_*/*U*_eq_	
H2	0.357 (2)	0.0915 (17)	0.0811 (19)	0.039 (7)*	
H9	0.5323 (18)	0.1563 (13)	0.5491 (16)	0.018 (5)*	
C1'	0.04998 (19)	0.36320 (14)	0.44410 (16)	0.0263 (4)	
H1'	0.0473	0.4182	0.4833	0.032*	
C2'	0.10902 (18)	0.28480 (14)	0.48503 (15)	0.0231 (4)	
H2'	0.1442	0.2879	0.5514	0.028*	
C2	0.37116 (16)	0.09545 (13)	0.23153 (13)	0.0180 (4)	
C3'	0.11662 (17)	0.20141 (13)	0.42843 (14)	0.0188 (4)	
C3	0.35748 (17)	−0.04166 (13)	0.11768 (14)	0.0204 (4)	
C4	0.33879 (19)	−0.07060 (14)	0.00610 (14)	0.0252 (4)	
H40	0.3814	−0.1287	−0.0061	0.038*	
H41	0.3706	−0.0225	−0.0386	0.038*	
H42	0.2524	−0.0790	−0.0083	0.038*	
C4'	0.05884 (17)	0.19924 (14)	0.33050 (14)	0.0216 (4)	
H4'	0.0611	0.1442	0.2913	0.026*	
C5	0.41102 (17)	0.13772 (12)	0.42180 (14)	0.0178 (4)	
C5'	−0.00180 (17)	0.27755 (14)	0.29079 (15)	0.0234 (4)	
H5'	−0.0408	0.2737	0.2260	0.028*	
C6	0.31344 (17)	0.12846 (13)	0.50691 (13)	0.0185 (4)	
H60	0.3370	0.0759	0.5512	0.022*	
H61	0.3176	0.1848	0.5495	0.022*	
C6'	−0.00551 (18)	0.36170 (14)	0.34573 (16)	0.0247 (4)	
C7	0.17835 (17)	0.11399 (13)	0.47324 (14)	0.0191 (4)	
H7	0.1752	0.0644	0.4203	0.023*	
C7'	−0.0625 (2)	0.44905 (15)	0.29915 (17)	0.0326 (5)	
H70'	−0.0004	0.4848	0.2644	0.049*	
H71'	−0.0974	0.4866	0.3533	0.049*	
H72'	−0.1260	0.4315	0.2500	0.049*	
C8	0.10620 (18)	0.07994 (13)	0.56829 (14)	0.0227 (4)	
H80	0.1403	0.0215	0.5928	0.034*	
H81	0.0213	0.0708	0.5487	0.034*	
H82	0.1120	0.1263	0.6226	0.034*	
C9	0.53585 (18)	0.15303 (13)	0.47409 (14)	0.0200 (4)	
C10	0.64558 (18)	0.16273 (13)	0.43130 (15)	0.0226 (4)	
C11	0.67035 (19)	0.16274 (15)	0.31623 (16)	0.0286 (5)	
H111	0.7302	0.1151	0.3009	0.043*	
H112	0.7013	0.2233	0.2961	0.043*	
H113	0.5953	0.1498	0.2784	0.043*	
C12	0.75806 (19)	0.17719 (15)	0.49976 (17)	0.0304 (5)	
H121	0.7982	0.2348	0.4807	0.046*	
H122	0.8138	0.1254	0.4911	0.046*	
H123	0.7339	0.1807	0.5712	0.046*	
C41	0.37297 (17)	0.30457 (13)	0.37021 (13)	0.0177 (4)	
C42	0.33714 (19)	0.37167 (13)	0.28539 (14)	0.0231 (4)	
H420	0.3991	0.3715	0.2330	0.035*	
H421	0.3296	0.4340	0.3137	0.035*	
H422	0.2597	0.3527	0.2550	0.035*	
N1	0.35903 (15)	0.05391 (11)	0.13421 (12)	0.0192 (3)	
N3	0.35840 (14)	0.18471 (10)	0.24065 (11)	0.0183 (3)	
N4	0.38013 (14)	0.21218 (10)	0.34384 (11)	0.0174 (3)	
O1	0.36850 (13)	−0.09778 (9)	0.18908 (10)	0.0256 (3)	
O2	0.39419 (12)	0.32905 (9)	0.46085 (9)	0.0213 (3)	
S1	0.40785 (4)	0.02872 (3)	0.34223 (3)	0.01914 (12)	

### Atomic displacement parameters (Å^2^)

**Table:**

	*U*^11^	*U*^22^	*U*^33^	*U*^12^	*U*^13^	*U*^23^
C1'	0.0305 (11)	0.0224 (10)	0.0261 (10)	0.0034 (8)	0.0050 (8)	−0.0035 (8)
C2'	0.0251 (10)	0.0248 (10)	0.0195 (9)	0.0012 (8)	0.0007 (8)	−0.0015 (8)
C2	0.0184 (9)	0.0205 (9)	0.0153 (9)	0.0010 (7)	0.0019 (7)	0.0011 (7)
C3'	0.0175 (9)	0.0199 (9)	0.0192 (9)	−0.0004 (7)	0.0020 (7)	0.0011 (7)
C3	0.0198 (9)	0.0198 (9)	0.0215 (10)	0.0000 (8)	0.0010 (7)	−0.0005 (7)
C4	0.0334 (11)	0.0220 (10)	0.0201 (10)	0.0008 (8)	−0.0041 (8)	−0.0041 (8)
C4'	0.0212 (10)	0.0228 (9)	0.0207 (9)	−0.0018 (8)	0.0007 (7)	−0.0010 (8)
C5	0.0221 (9)	0.0169 (9)	0.0144 (8)	0.0007 (7)	0.0003 (7)	0.0003 (7)
C5'	0.0206 (10)	0.0304 (10)	0.0192 (9)	−0.0002 (8)	0.0002 (8)	0.0043 (8)
C6	0.0239 (10)	0.0187 (9)	0.0128 (8)	0.0013 (8)	−0.0008 (7)	0.0012 (7)
C6'	0.0196 (10)	0.0272 (10)	0.0276 (10)	0.0032 (8)	0.0058 (8)	0.0068 (8)
C7	0.0231 (10)	0.0181 (9)	0.0160 (9)	−0.0001 (7)	0.0011 (7)	−0.0020 (7)
C7'	0.0328 (12)	0.0314 (11)	0.0338 (12)	0.0119 (9)	0.0082 (9)	0.0077 (9)
C8	0.0248 (10)	0.0216 (9)	0.0216 (10)	−0.0014 (8)	0.0009 (8)	0.0000 (8)
C9	0.0245 (10)	0.0197 (9)	0.0157 (9)	0.0011 (7)	−0.0032 (7)	0.0018 (7)
C10	0.0253 (10)	0.0171 (9)	0.0254 (10)	0.0020 (8)	−0.0015 (8)	0.0013 (7)
C11	0.0258 (11)	0.0285 (11)	0.0316 (11)	−0.0001 (9)	0.0064 (9)	−0.0002 (9)
C12	0.0229 (11)	0.0271 (11)	0.0410 (12)	0.0019 (9)	−0.0029 (9)	−0.0013 (9)
C41	0.0193 (9)	0.0187 (9)	0.0153 (9)	−0.0014 (7)	0.0009 (7)	−0.0003 (7)
C42	0.0332 (11)	0.0183 (9)	0.0176 (9)	0.0004 (8)	−0.0029 (8)	−0.0003 (7)
N1	0.0274 (9)	0.0185 (8)	0.0117 (7)	0.0001 (7)	0.0000 (6)	−0.0010 (6)
N3	0.0230 (8)	0.0188 (8)	0.0130 (7)	0.0003 (6)	−0.0009 (6)	−0.0019 (6)
N4	0.0240 (8)	0.0174 (7)	0.0107 (7)	0.0021 (6)	−0.0006 (6)	0.0014 (6)
O1	0.0358 (8)	0.0201 (7)	0.0208 (7)	0.0013 (6)	0.0010 (6)	0.0016 (6)
O2	0.0277 (7)	0.0211 (7)	0.0149 (6)	0.0000 (6)	−0.0011 (5)	−0.0019 (5)
S1	0.0254 (2)	0.0175 (2)	0.0145 (2)	0.00277 (19)	0.00084 (17)	0.00137 (18)

### Geometric parameters (Å, °)

**Table:**

C1'—C2'	1.384 (3)	C6'—C7'	1.505 (3)
C1'—C6'	1.392 (3)	C7—C8	1.540 (3)
C1'—H1'	0.93	C7—H7	0.98
C2'—C3'	1.392 (3)	C7'—H70'	0.96
C2'—H2'	0.93	C7'—H71'	0.96
C2—N3	1.280 (2)	C7'—H72'	0.96
C2—N1	1.387 (2)	C8—H80	0.96
C2—S1	1.7497 (18)	C8—H81	0.96
C3'—C4'	1.397 (3)	C8—H82	0.96
C3'—C7	1.519 (2)	C9—C10	1.327 (3)
C3—O1	1.219 (2)	C9—H9	0.97 (2)
C3—N1	1.373 (2)	C10—C12	1.507 (3)
C3—C4	1.502 (3)	C10—C11	1.508 (3)
C4—H40	0.96	C11—H111	0.96
C4—H41	0.96	C11—H112	0.96
C4—H42	0.96	C11—H113	0.96
C4'—C5'	1.385 (3)	C12—H121	0.96
C4'—H4'	0.93	C12—H122	0.96
C5—N4	1.491 (2)	C12—H123	0.96
C5—C9	1.518 (3)	C41—O2	1.234 (2)
C5—C6	1.541 (2)	C41—N4	1.357 (2)
C5—S1	1.8545 (18)	C41—C42	1.494 (2)
C5'—C6'	1.388 (3)	C42—H420	0.96
C5'—H5'	0.93	C42—H421	0.96
C6—C7	1.536 (3)	C42—H422	0.96
C6—H60	0.97	N1—H2	0.87 (2)
C6—H61	0.97	N3—N4	1.399 (2)
			
C2'—C1'—C6'	121.60 (18)	C6'—C7'—H70'	109.5
C2'—C1'—H1'	119.2	C6'—C7'—H71'	109.5
C6'—C1'—H1'	119.2	H70'—C7'—H71'	109.5
C1'—C2'—C3'	121.06 (18)	C6'—C7'—H72'	109.5
C1'—C2'—H2'	119.5	H70'—C7'—H72'	109.5
C3'—C2'—H2'	119.5	H71'—C7'—H72'	109.5
N3—C2—N1	119.63 (16)	C7—C8—H80	109.5
N3—C2—S1	119.00 (14)	C7—C8—H81	109.5
N1—C2—S1	121.35 (14)	H80—C8—H81	109.5
C2'—C3'—C4'	117.38 (17)	C7—C8—H82	109.5
C2'—C3'—C7	121.72 (16)	H80—C8—H82	109.5
C4'—C3'—C7	120.80 (16)	H81—C8—H82	109.5
O1—C3—N1	121.87 (17)	C10—C9—C5	129.19 (17)
O1—C3—C4	123.36 (17)	C10—C9—H9	117.4 (12)
N1—C3—C4	114.76 (16)	C5—C9—H9	113.5 (12)
C3—C4—H40	109.5	C9—C10—C12	119.73 (18)
C3—C4—H41	109.5	C9—C10—C11	125.62 (18)
H40—C4—H41	109.5	C12—C10—C11	114.63 (17)
C3—C4—H42	109.5	C10—C11—H111	109.5
H40—C4—H42	109.5	C10—C11—H112	109.5
H41—C4—H42	109.5	H111—C11—H112	109.5
C5'—C4'—C3'	121.18 (18)	C10—C11—H113	109.5
C5'—C4'—H4'	119.4	H111—C11—H113	109.5
C3'—C4'—H4'	119.4	H112—C11—H113	109.5
N4—C5—C9	112.66 (15)	C10—C12—H121	109.5
N4—C5—C6	112.84 (14)	C10—C12—H122	109.5
C9—C5—C6	108.48 (15)	H121—C12—H122	109.5
N4—C5—S1	102.63 (11)	C10—C12—H123	109.5
C9—C5—S1	111.83 (13)	H121—C12—H123	109.5
C6—C5—S1	108.29 (12)	H122—C12—H123	109.5
C4'—C5'—C6'	121.37 (18)	O2—C41—N4	119.83 (16)
C4'—C5'—H5'	119.3	O2—C41—C42	123.49 (16)
C6'—C5'—H5'	119.3	N4—C41—C42	116.67 (15)
C7—C6—C5	118.41 (15)	C41—C42—H420	109.5
C7—C6—H60	107.7	C41—C42—H421	109.5
C5—C6—H60	107.7	H420—C42—H421	109.5
C7—C6—H61	107.7	C41—C42—H422	109.5
C5—C6—H61	107.7	H420—C42—H422	109.5
H60—C6—H61	107.1	H421—C42—H422	109.5
C5'—C6'—C1'	117.35 (18)	C3—N1—C2	124.06 (16)
C5'—C6'—C7'	121.43 (18)	C3—N1—H2	119.1 (16)
C1'—C6'—C7'	121.17 (19)	C2—N1—H2	116.7 (16)
C3'—C7—C6	114.25 (15)	C2—N3—N4	110.24 (14)
C3'—C7—C8	109.29 (15)	C41—N4—N3	119.79 (14)
C6—C7—C8	108.32 (15)	C41—N4—C5	122.03 (14)
C3'—C7—H7	108.3	N3—N4—C5	118.18 (14)
C6—C7—H7	108.3	C2—S1—C5	89.94 (8)
C8—C7—H7	108.3		
			
C6'—C1'—C2'—C3'	0.9 (3)	O1—C3—N1—C2	−1.0 (3)
C1'—C2'—C3'—C4'	−2.1 (3)	C4—C3—N1—C2	177.88 (17)
C1'—C2'—C3'—C7	−178.47 (17)	N3—C2—N1—C3	−172.52 (18)
C2'—C3'—C4'—C5'	1.0 (3)	S1—C2—N1—C3	9.3 (3)
C7—C3'—C4'—C5'	177.45 (17)	N1—C2—N3—N4	−177.12 (15)
C3'—C4'—C5'—C6'	1.3 (3)	S1—C2—N3—N4	1.1 (2)
N4—C5—C6—C7	53.5 (2)	O2—C41—N4—N3	−178.76 (15)
C9—C5—C6—C7	179.04 (15)	C42—C41—N4—N3	2.1 (2)
S1—C5—C6—C7	−59.41 (18)	O2—C41—N4—C5	1.1 (3)
C4'—C5'—C6'—C1'	−2.5 (3)	C42—C41—N4—C5	−178.05 (16)
C4'—C5'—C6'—C7'	175.03 (18)	C2—N3—N4—C41	179.12 (16)
C2'—C1'—C6'—C5'	1.4 (3)	C2—N3—N4—C5	−0.7 (2)
C2'—C1'—C6'—C7'	−176.10 (19)	C9—C5—N4—C41	−59.4 (2)
C2'—C3'—C7—C6	−57.9 (2)	C6—C5—N4—C41	63.9 (2)
C4'—C3'—C7—C6	125.84 (18)	S1—C5—N4—C41	−179.78 (14)
C2'—C3'—C7—C8	63.6 (2)	C9—C5—N4—N3	120.51 (17)
C4'—C3'—C7—C8	−112.64 (19)	C6—C5—N4—N3	−116.23 (16)
C5—C6—C7—C3'	−73.3 (2)	S1—C5—N4—N3	0.08 (18)
C5—C6—C7—C8	164.63 (15)	N3—C2—S1—C5	−0.96 (16)
N4—C5—C9—C10	−55.8 (3)	N1—C2—S1—C5	177.27 (16)
C6—C5—C9—C10	178.55 (19)	N4—C5—S1—C2	0.41 (12)
S1—C5—C9—C10	59.2 (2)	C9—C5—S1—C2	−120.59 (14)
C5—C9—C10—C12	−179.85 (17)	C6—C5—S1—C2	119.95 (13)
C5—C9—C10—C11	1.5 (3)		

### Hydrogen-bond geometry (Å, °)

**Table:**

*D*—H···*A*	*D*—H	H···*A*	*D*···*A*	*D*—H···*A*
N1—H2···O2^i^	0.87 (2)	1.96 (2)	2.811 (2)	167 (2)

Symmetry codes: (i) *x*, −*y*+1/2, *z*−1/2.
